# The footprint of metabolism in the organization of mammalian genomes

**DOI:** 10.1186/1471-2164-13-174

**Published:** 2012-05-08

**Authors:** Luisa Berná, Ankita Chaurasia, Claudia Angelini, Concetta Federico, Salvatore Saccone, Giuseppe D'Onofrio

**Affiliations:** 1 Genome Evolution and Organization - Department Animal Physiology and Evolution, Stazione Zoologica Anton Dohrn, Villa Comunale, 80121 Naples, Italy; 2 Sección Biomatematica, Facultad de Ciencias, Universidad de la República, Iguá 4225, Montevideo, Uruguay; 3 Istituto per le Applicazioni del Calcolo "Mauro Picone", IAC-CNR, Via Pietro Castellino, 111-80131 Naples, Italy; 4 Dipartimento di Scienze Biologiche, Geologiche e Ambientali, Università di Catania Sezione Biologia.Animale "M. La Greca", Via Androne, 81-95124 Catania, Italy

## Abstract

**Background:**

At present five evolutionary hypotheses have been proposed to explain the great variability of the genomic GC content among and within genomes: the mutational bias, the biased gene conversion, the DNA breakpoints distribution, the thermal stability and the metabolic rate. Several studies carried out on bacteria and teleostean fish pointed towards the critical role played by the environment on the metabolic rate in shaping the base composition of genomes. In mammals the debate is still open, and evidences have been produced in favor of each evolutionary hypothesis. Human genes were assigned to three large functional categories (as well as to the corresponding functional classes) according to the KOG database: (i) information storage and processing, (ii) cellular processes and signaling, and (iii) metabolism. The classification was extended to the organisms so far analyzed performing a reciprocal Blastp and selecting the best reciprocal hit. The base composition was calculated for each sequence of the whole CDS dataset.

**Results:**

The GC3 level of the above functional categories was increasing from (i) to (iii). This specific compositional pattern was found, as footprint, in all mammalian genomes, but not in frog and lizard ones. Comparative analysis of human versus both frog and lizard functional categories showed that genes involved in the metabolic processes underwent the highest GC3 increment. Analyzing the KOG functional classes of genes, again a well defined intra-genomic pattern was found in all mammals. Not only genes of metabolic pathways, but also genes involved in chromatin structure and dynamics, transcription, signal transduction mechanisms and cytoskeleton, showed an average GC3 level higher than that of the whole genome. In the case of the human genome, the genes of the aforementioned functional categories showed a high probability to be associated with the chromosomal bands.

**Conclusions:**

In the light of different evolutionary hypotheses proposed so far, and contributing with different potential to the genome compositional heterogeneity of mammalian genomes, the one based on the metabolic rate seems to play not a minor role. Keeping in mind similar results reported in bacteria and in teleosts, the specific compositional patterns observed in mammals highlight metabolic rate as unifying factor that fits over a wide range of living organisms.

## Background

As recently stated by Meyer and collaborators "structure and organization of genomes belongs among the key questions of genome biology" [1]. One of the most crucial and largely debated questions is centered on the nature of the forces driving the base compositional variation among genomes. At present as much as five evolutionary hypotheses have been proposed to explain the great variability of the genomic GC content, which can be split in two groups, on the bases of the nature of the forces driving the genome evolution, *i.e*. intra- or extra-cellular [2]. The former, including the mutational bias, the biased gene conversion (BGC) and the DNA breakpoints distribution (BPR) hypotheses, is mainly founded on stochastic events arising during intracellular processes, such as DNA replication, repair and recombination. The latter, including the thermal stability and the metabolic rate hypotheses, take into account the role of adaptive processes resulting from the interaction of the organism with the surrounding environment.

In the frame of the neutral theory, the mutational bias hypothesis [3-5] was first proposed to explain the great variation of the genomic GC content among bacteria, and later on extended to higher vertebrates [6]. In the same frame, the BGC is based essentially on a synergy between recombination events and biased DNA repair system [7-9]. The BPR hypothesis considers that evolutionary rearrangement of breakages happen with a uniform propensity along the genome. Growing body of evidence shows a heterogeneous distribution of breakpoints in mammalian genomes, occurring more frequently in the GC-rich regions, harboring replication origin sites and characterized by high transcriptional activity [10].

In the frame of the adaptive point of view, several environmental factors significantly correlated with the DNA base composition, have been reported in bacteria: competition for metabolic resources [11], anaerobiosis [12], endosymbiosis [13], environments/habitats [2], growth temperature [14], and "aerobic respiration" [15]. In particular, the last two papers stressed the effect on the genomic GC content of the main factors affecting the environmental dynamics: temperature and metabolism. According to the thermodynamic hypothesis, an increment of environmental or body temperature triggers a GC increment, stabilizing DNA, RNA and proteins [16]. The hypothesis based on the metabolic rate was grounded on two DNA features, bendability [17] and nucleosome formation potential [18], both significantly correlated with the GC content. More precisely, a higher DNA bendability and a decrement of the nucleosome formation potential have been reported to be both favored by an increment of the GC content [17,18]. Accordingly, GC-richest genomic regions showed a high transcriptional activity [19,20].

Preliminary analysis of human genes grouped in functional classes according to the KOG database [21,22] showed a biased distribution of the GC3 level that was significantly higher in the functional classes of genes involved in metabolic processes [23]. In the present paper, the analysis of the KOG categories and functional classes of genes was extended to thirteen completely sequenced mammalian genomes, as well as to amphibian (*X. tropicalis*) and reptile (*A. carolinensis*) genomes.

Current results confirmed previous conclusions [23], further stressing the role of metabolic rate in shaping the mammalian genome organization. Indeed, a compositional hierarchy among functional categories was found, and the GC3 content of the genes involved in metabolic processes was the highest in all mammalian genomes so far analyzed. Interestingly, the mammalian compositional pattern was absent in the amphibian and the reptile genomes. The finding opened critical evolutionary questions on the compositional transition from "cold- to warm-blooded vertebrates" [24-26], more precisely from amphibian/reptile to mammals, and will be discussed in the light of the current hypotheses about genome compositional variability.

Interestingly, in all mammals the functional classes that recurrently showed a GC3 level higher than that of the whole genome were, apart from those involved in metabolic processes, those involved in: Chromatin structure and dynamics, Transcription, Signal transduction mechanisms and Cytoskeleton. In the human genome, the aforesaid functional classes showed a high probability to cluster in the GC-richest chromosomal bands. In the light of current literature this organization could reflect the needs of a coordinated response to stressing stimuli altering the normal metabolic rate.

## Methods

In the KOG database http://www.ncbi.nlm.nih.gov/COG/[21,22] human proteins were grouped in functional classes (denoted by capital letters in square brackets), in turn grouped in three large categories, namely: (i) information storage and processing; (ii) cellular processes and signaling; (iii) metabolism. The corresponding protein and coding sequences (CDS) were retrieved from NCBI http://www.ncbi.nlm.nih.gov using a batch entrez function. Proteins classified in more than one class were removed from further analysis. Genes whose function was predicted only [R] or unknown [S], representing about 19% of the all dataset were removed from further analyses. In order to avoid statistical bias, the functional classes represented by less than a hundred sequences, namely [M], [N] and [Y], accounting overall for less than one percent of the all dataset, were also removed. For sake of simplicity square brackets denoting the functional classes were not used in the other sections of the present paper.

The whole set of human CDS, as well as that of the following species (in alphabetical order): *Anolis carolinensis, Bos taurus, Dasypus novemcinctus, Equus caballus, Gorilla gorilla, Loxodonta africana, Monodelphis domestica, Mus musculus, Ornithorhynchus anatinus, Oryctolagus cuniculus, Pteropus vampyrus, Pongo pygmaeus, Spermophilus tridecemlineatus, Tursiops truncatus *and *Xenopus tropicalis *were retrieved from the Ensemble database http://www.ensembl.org. CDS were classified in the KOG functional classes through the orthology with the human proteins. In other words, for each mammalian genome each gene acquired the same KOG classification of the corresponding human gene after the identification of the orthologous pair. In order to identify orthologs, a Perl script, essentially performing reciprocal Blastp [27] (e-value < 1e-05) and selecting the best reciprocal hit (BRH), was compiled.

Flanking regions (2000 bp flanking at 5'and 3' the transcript) and intronic sequences of KOG human genes were retrieved respectively form Ensemble http://www.ensembl.org using Biomart tools, and from UCSC http://genome.ucsc.edu/.

CodonW (1.4.4) was used to calculate the GC content (*i.e*. the molar ratio of guanine plus cytosine) of coding and non-coding sequences, as well as the GC3 content (*i.e*. the molar ratio of guanine plus cytosine the third codon positions) of CDS. The average of GC3 level was calculated for each sequence of each genome so far analyzed. In order to determine the statistical significance of the differences in GC3 content between the three main categories of genes, a two-tale Mann-Whitney test was performed.

The **de Finetti's **diagram was used to assess the compositional/spatial distribution of the three categories in different genomes (see Additional file 1 for a detailed description of the analysis). Shortly, for each organism the whole GC3 range was split in three intervals of equal size, denoted as Low, Medium and High, respectively. The number of functional classes belonging to the three categories were counted in each interval and normalized to 1 for plotting.

For each species the average GC3 level of each functional class was compared with that of the genome (*i.e*. the average of the GC3 level calculated using all the available coding sequences of the species), and statistical significance was assessed by the t-Student's test, with Bonferroni's correction (α = 0.05) for multiple-comparisons. The data were showed as **Butterfly plot**.

Human CDS from each KOG class were assigned to the different compositional band types (L1+, L1-, H3-, H3+ bands) previously identified in the human chromosomes [28,29].

## Results

**Figure 1:**
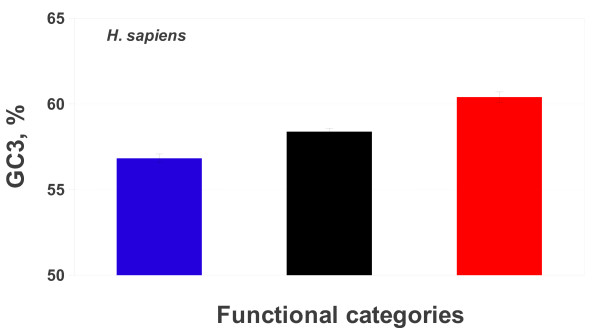
**The histogram shows the average GC3 content in the three functional categories of the human genome: (i) information storage and processing (Blue bar); (ii) cellular processes and signaling (Black bar); (iii) metabolism (Red bar)**. For each histogram bar the Standard error is reported.

In the KOG database [21,22] human genes were classified in 25 functional classes, denoted by capital letters in square brackets, and grouped in three main categories: i) information storage and processing, represented by five functional classes; ii) cellular processes and signaling, represented by ten functional classes; and iii) metabolism, represented by eight functional classes. The three categories, from now on denoted as Blue, Black, and Red for sake of simplicity, accounted, respectively, for 22%, 42% and 16% of the all KOG dataset. The complete list of the functional classes, the corresponding number of genes and the average GC3 level of each functional class were reported in Table 1. The average GC3 content of the human genome (58.5%) was very close to that of the KOG dataset (58.4%). Within the human genome, the average GC3 of the three categories was significantly different (Figure 1). Indeed, the GC3 of Red category was significantly higher than that of both Black and Blue ones (*p *< 2.2 × 10^-16 ^and *p *< 4.2 × 10^-8^, respectively). In turn, the GC3 of the Black category was significantly higher than that of Blue one (*p *< 1.6 × 10^-6^). In short, in the human genome the GC3 content of the three functional categories showed the following trend: Blue < Black < Red (Figure 1).

**Table 1:** Functional classification of human genes

KOG classes	Categories	#	GC3	**s.d**.
	**INFORMATION STORAGE AND PROCESSING**			

[A]	RNA processing and modification	600	0.517	0.151

[B]	Chromatin structure and dynamics	224	0.610	0.172

[J]	Translation, ribosomal structure and biogenesis	1273	0.545	0.117

[K]	Transcription	1137	0.619	0.170

[L]	Replication, recombination and repair	300	0.546	0.154

	**CELLULAR PROCESSES AND SIGNALING**			

[D]	Cell cycle control, cell division, chromosome partitioning	267	0.552	0.160

[M]	Cell wall/membrane/envelope biogenesis	63	0.576	0.164

[N]	Cell motility	26	0.586	0.163

[O]	Posttranslational modification, protein turnover, chaperones	1471	0.557	0.151

[T]	Signal transduction mechanisms	2214	0.616	0.158

[U]	Intracellular trafficking, secretion, and vesicular transport	685	0.571	0.161

[V]	Defense mechanisms	1023	0.527	0.172

[W]	Extracellular structures	284	0.588	0.150

[Y]	Nuclear structure	17	0.534	0.150

[Z]	Cytoskeleton	801	0.638	0.158

	**METABOLISM**			

[C]	Energy production and conversion	403	0.576	0.153

[E]	Amino acid transport and metabolism	499	0.618	0.154

[F]	Nucleotide transport and metabolism	187	0.588	0.151

[G]	Carbohydrate transport and metabolism	469	0.606	0.150

[H]	Coenzyme transport and metabolism	102	0.563	0.135

[I]	Lipid transport and metabolism	410	0.595	0.154

[P]	Inorganic ion transport and metabolism	402	0.646	0.161

[Q]	Secondary metabolites biosynthesis, transport and catabolism	191	0.591	0.155

	**POORLY CHARACTERIZED**			

[R]	General function prediction only	1889	0.593	0.162

[S]	Function unknown	1171	0.568	0.162

	**Total number of genes**	**16118**	**0.584**	**0.160**

(#) Number of genes

**Figure 2:**
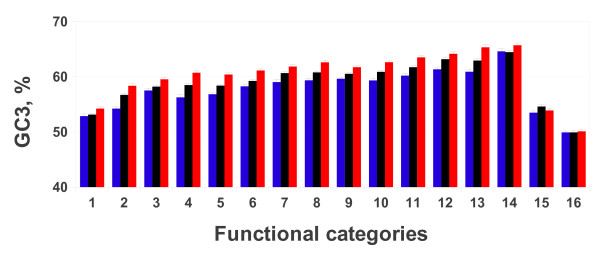
**The histogram shows the average GC3 content in the three functional categories in all analyzed genomes**. Color code of histogram bars is as in Figure 1. For each histogram bar standard error is reported. Genome legend: *M. domestica *(1), *S. tridecemlineatus *(2), *L. africana *(3), *D. novemcinctus *(4), *H. sapiens *(5), *P. pygmaeus *(6), *P. vampyrus *(7), *E. caballus *(8), *M. musculus *(9), *G. gorilla *(10), *T. truncatus *(11), *B. taurus *(12), *O. cuniculus *(13), *O. anatinus *(14), *A. carolinensis *(15) and *X. tropicalis *(16).

Using the best reciprocal hit (BRH) approach the genes of mammalian genomes representing the following orders: **primates **(*G. gorilla *and *P. pygmaeus*), **rodentia **(*M. musculus*, and *S. tridecemlineatus*), **lagomorpha (***O. cuniculus***) artiodactyla **(*B. taurus*) **perissodactyla **(*E. caballus*), **chiroptera **(*P. vampyrus*), **cetacea **(*T. truncatus*), **proboscidea **(*L. africana*), **cingulata **(*D. novemcinctus*), **didelphimorphia **(*M. domestica*) and **monotremata **(*O. anatinus*) were classified in the KOG functional classes through the orthology with the human ones. The same approach was used to classify the genes of the species representing the order of **anura **(*X. tropicalis*) and **squamata **(*A. carolinensis*) genes. For each genome the whole number of available genomic CDS, the subset of genes classified according to the KOG database, the amount of genes belonging to the Blue, Black and Red categories, as well as the corresponding GC3 levels and the standard deviation, were reported in Table 2. Interestingly, the same trend of the GC3 content observed in the human genome, *i.e*. Blue < Black < Red, was found in almost all mammalian genomes. In the case of both platypus (*M. domestica*) and opossum (*O. anatinus*) genomes (Figure 2) the GC3 content of the Red category was significantly the highest, but no significant differences were observed comparing the GC3 content of the Black and the Blue categories (see Additional file 2).

**Table 2:** Average GC3 levels, standard deviation and gene number of KOG's functional categories

		KOG**			BLUE*			BLACK*			RED*		
	**Organism**	**GC3**	**s.d**.	**#**	**GC3**	**s.d**.	**#**	**GC3**	**s.d**.	**#**	**GC3**	**s.d**.	**#**

Mammals	*H. sapiens*	0.584	0.159	12942	0.568	0.154	3564	0.584	0.163	6745	0.604	0.155	2663

	*G. Gorilla*	0.609	0.162	6268	0.593	0.166	1491	0.609	0.166	3357	0.626	0.148	1420

	*P. pygmaeus*	0.594	0.164	7455	0.583	0.167	1766	0.593	0.166	4012	0.611	0.154	1677

	*M. musculus*	0.606	0.114	7505	0.596	0.127	1780	0.605	0.122	4032	0.617	0.101	1693

	*O. cuniculus*	0.630	0.175	5413	0.609	0.179	1296	0.629	0.177	2867	0.653	0.164	1250

	*S. tridecemilineatus*	0.565	0.154	5455	0.542	0.157	1325	0.567	0.155	2900	0.584	0.144	1230

	*B. taurus*	0.630	0.167	7139	0.613	0.171	1706	0.632	0.169	3794	0.642	0.155	1639

	*E. caballus*	0.609	0.164	7102	0.594	0.170	1646	0.608	0.165	3840	0.626	0.153	1613

	*P. vampyrus*	0.605	0.164	6780	0.590	0.170	1638	0.607	0.165	3607	0.619	0.155	1535

	*T. truncatus*	0.618	0.167	6812	0.602	0.172	1635	0.617	0.169	3634	0.635	0.155	1543

	*L. africana*	0.583	0.152	5704	0.575	0.157	1413	0.582	0.153	3007	0.595	0.141	1284

	*D. novemcinctus*	0.585	0.180	5358	0.563	0.181	1310	0.585	0.182	2832	0.607	0.173	1216

	*O. anatinus*	0.648	0.166	5287	0.646	0.169	1319	0.646	0.166	2734	0.657	0.161	1234

	*M. domestica*	0.533	0.145	3598	0.529	0.153	1641	0.531	0.144	3598	0.542	0.137	1578

Reptiles	*A. cardiensis*	0.539	0.159	5959	0.535	0.158	3063	0.546	0.1655	1498	0.539	0.153	1398
Amphibians	*X. tropicalis*	0.500	0.112	3584	0.499	0.112	1753	0.499	0.1174	961	0.501	0.108	870

(*) Blue, Black and Red refers to the gene classification of KOG database (see Methods)

(**) Genes orthologous to KOG human genes

(#) Number of genes

In the amphibian and reptile genomes the GC3 content of the Red category was never significantly different from that of the Black and Blue categories, thus the mammalian pattern (Blue < Black < Red) was not observed (Figure 2 and Additional file 2).

**Figure 3:**
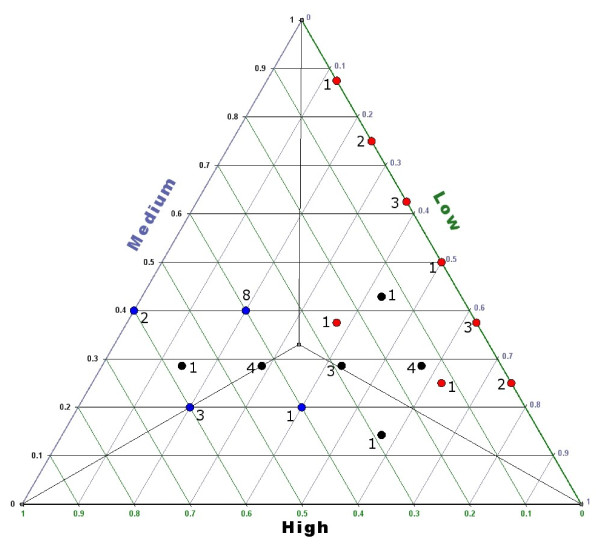
**de Finetti's diagram shows the spatial distribution of the three functional categories: (i) information storage and processing (Blue dots); (ii) cellular processes and signaling (Black dots); (iii) metabolism (Red dots)**. **Numbers close to dots refer to the occurrence of overlapping genomes**.

In order to compare across the different genomes the impact of the Blue < Black < Red relation, a descriptive analysis of the distribution of the functional categories over a GC3 range was performed. The results were reported in the **de Finetti's **diagram (Figure 3), showing the distance of a given point from a given side accounting for the frequency of a given category in one of the three GC3 ranges, namely Low, Medium and High. Obviously, short distances accounted for low frequencies. In the diagram: i) the Red category was rarely present in the Low GC3 range, being mainly confined to a restricted part of the diagram space, *i.e*. the upper right side; ii) no overlap was observed between the spatial distribution of the Red with that of the Blue category; and iii) partial overlap was observed between the Blue and Black categories which did not show specific distribution, although rarely present in the High GC3 range. The significance of assumption was tested performing 1000 class permutations and observing the diagram distribution of the red classes (see Additional file 1). The probability to reproduce by chance the confinement of the Red category in the upper right side of the diagram was estimate to be *p *< 1.76 × 10^-2^. In contrast to mammals, the spatial distribution of the three categories was not significantly different in frog and lizard genomes (data not shown).

**Figure 4:**
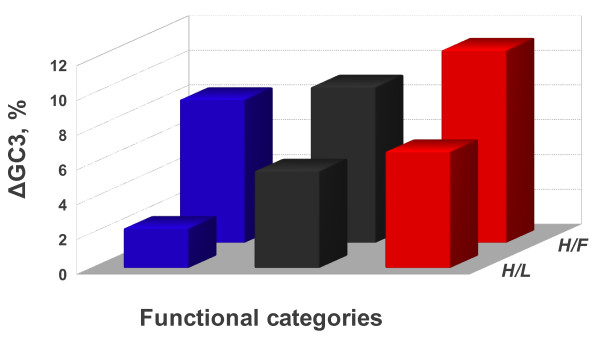
**The histogram shows the average GC3 increment in the three functional categories comparing human vs frog *(H/F) *and human vs lizard *(H/L)***. Color code of histogram bars is as in Figure 1. In the H/F comparison the standard error for the Blue, Black and Red bars were, respectively, 5.1 × 10^-3^, 3.7 × 10^-3 ^and 5.5 × 10^-3^. In the H/L comparison the standard error for the Blue, Black and Red bars were, respectively, 3.8 × 10^-3^, 2.8 × 10^-3 ^and 3.9 × 10^-3^.

A comparative compositional analysis of the functional categories between human and frog (H/F), and between human and lizard (H/L) was performed. More precisely the GC3 increment (&#916GC3) was investigated. In both comparisons, positive values of ΔGC3 were observed in the human categories (Figure 4). Interestingly, in both H/F and H/L comparisons the highest &#916GC3 increment was observed in the Red category. Infact, &#916GC3 of the Red category was significantly higher than that of the Black (*p *< 4.72 × 10^-4 ^and *p *< 1.14 × 10^-2^, respectively for the H/F and H/L), and significantly higher than that of the Blue category (*p *< 4.56 × 10^-5 ^and *p *< 2.27 × 10^-16^, respectively for the H/F and H/L).

**Figure 5:**
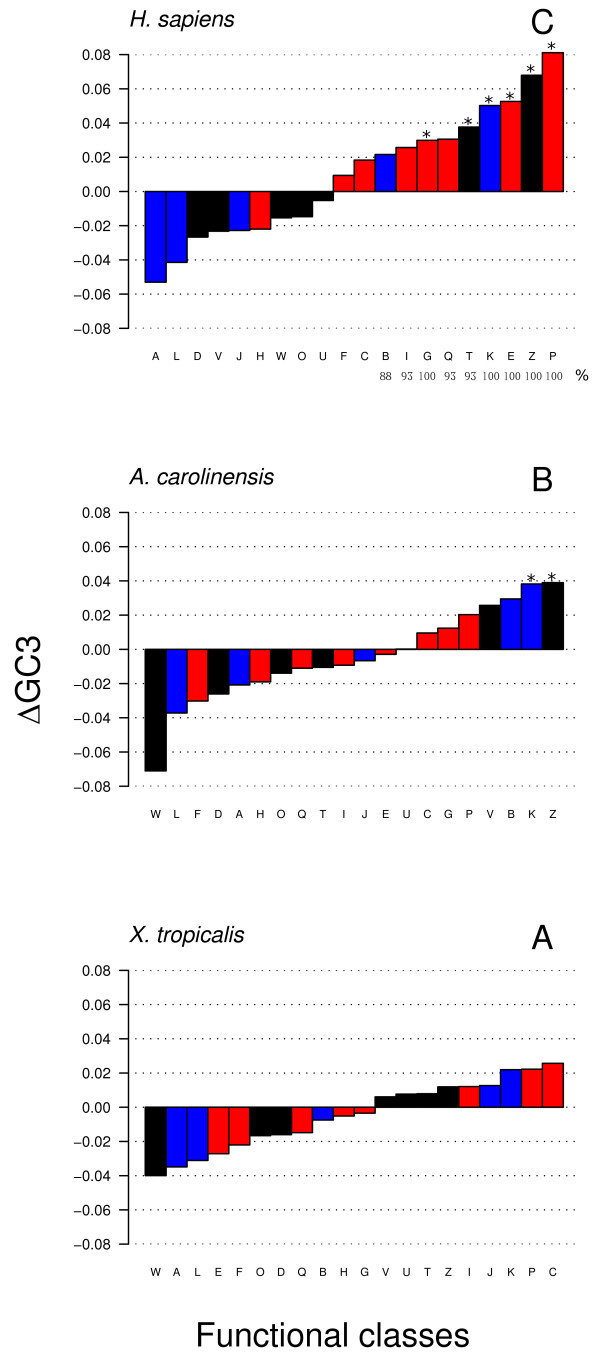
**The butterfly plot of frog (panel A), lizard (panel B) and human functional classes (panel C)**. Color code of histogram bars is as in Figure 1. Asterisks show bars with an average GC3 content significantly higher than the genomic one (Bonferroni's test, α = 0.05).

In order to shed light on the genome organization of the species so far analyzed, within each genome the average GC3 of each KOG functional class (see Table 1 for detailed definition) was investigated. More precisely, the difference between the GC3 levels of each functional class against that of the corresponding genome was calculated. Clustering negative and positive values a so-called butterfly plot for each genome was obtained. An overview of the all butterfly plots and detailed representations of each genome were reported in (Additional files 3, 4, 5, 6, 7). At first glance, the butterfly plots of the mammalian genomes showed an unbalance distribution of the bars (Additional files 3, 4, 5, 6, 7). Indeed, the bars of the Blue and Black categories where mainly in the negative side of the histogram, whereas those of the Red category where mainly in the positive side. In human, for instance, only two over five blue classes (namely B and K) and three the over seven black classes (namely T and Z) were in the positive side of the butterfly plot (Figure 5, panel C). On the contrary, six over eight Red classes (namely F, C, I, G, Q, E and P) were in the positive side of the butterfly plot (Figure 5, panel C). This picture was recurrently found among mammalian genomes (Additional files 3, 4, 5, 6, 7). The B functional class, for instance, was in the positive side in 88% of the mammalian genomes, whereas the classes G, K, E, Z and P were in the positive side of the butterfly plot in 100% of the cases (Figure 5, panel C). A t-Student's test with Bonferroni's correction (α = 0.05) was performed. The following functional classes: K (Blue), T and Z (Black) and G, E and P (Red), turned out to have an average GC3 level significantly higher than that of the whole genome (labeled by an asterisk in Figure 5, panel C). The butterfly plots of *X. tropicalis *and *A. carolinensis *were also reported (Figure 5; panel A and B, respectively). Regarding the reptile genome only two functional classes, namely the K and the Z classes showed an average GC3 level significantly higher than the genomic one (Figure 5, panel B), while none of the frog functional classes turned out to be significantly different (Figure 5, panel A).

**Figure 6:**
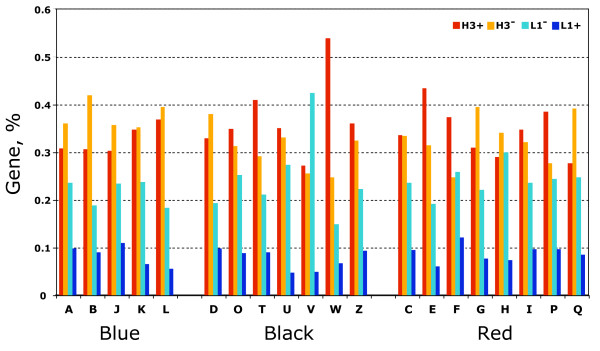
**The histogram shows the gene frequency in the four types of human chromosomal bands for each KOG functional class of the Blue, Black and Red KOG categories**.

The distribution of the human genes of each KOG class in the four chromosomal band types, namely L1+, L1-, H3- and H3+, characterized by increasing GC content [29], was analyzed (Figure 6). As general rule, the gene frequency was increasing as the GC content of the chromosomal band type was increasing. However, regarding the genes belonging to the Blue category, the maximum of the gene frequency was observed in the H3- bands, whereas that of Black and Red categories was in the H3+ (Figure 6). The χ^2 ^- test showed an association between the three functional categories (Blue, Black and Red) and the two chromosomal bands types (H3- and H3+) far from the randomness, *p *< < 10^-10^. Moreover, using the one side Z-test as test for proportion it was found that: i) regarding the Blue category, the gene frequency of the functional classes B and J was significantly higher in H3- (*p *< 2.8 × 10^-2 ^and *p *< 2.4 × 10^-2^, respectively), that of class A showed the same trend (but at the limit of significance, *p *< 7.0 × 10^-2^), whereas the functional classes K and L, although having the same trend, did not show significant *p*-values; ii) regarding the Black category, three out of seven functional classes, namely T, W and O, were significantly higher in the H3+ bands (*p *< 2.8 × 10^-10^, *p *< 1.0 × 10^-6 ^and *p *< 5.6 × 10^-2^, respectively), whereas the remaining classes showed no trend; finally iii) regarding the Red category, five out of eight functional classes showed a significantly different distribution between the chromosomal bands H3- and H3+, but a non uniform trend was observed. More precisely, the functional classes E, F and P were significantly higher in H3+ (*p *< 1.8 × 10^-3^, 1.9 × 10^-2 ^and 5.5 × 10^-3^, respectively), while G and Q showed opposite trend (*p *< 1.7 × 10^-2 ^and 3.5 × 10^-2^, respectively). Dividing the functional classes in two groups, *i.e*. positive and negative, according to the position of the corresponding functional class in the butterfly plot of Figure 5 (panel C), small differences were found between the two groups in the L1+ and L1- bands (about 2%), whereas in the H3- and H3+ bands the differences increased up to 5% and 7%, respectively. In short, the probability to find genes of the positive group in the H3+ bands was significantly higher than in the L1+ bands (*Z*-test one tail, *p *< 10^-2^).

## Discussion

Since the pioneering Ikemura's papers [30,31], the GC3 content, accounting for the base composition at the third (wobble) position in a codon, has been generally associated mainly with the codon usage and with the tRNA content. Furthermore, studies primarily performed on the human genome, showed that GC3 should be considered a keystone parameter to understand genome evolution. Indeed, GC3 turned out to be significantly correlated with the amino acid frequencies, *i.e*. GC1 + 2, as well as with the GC content of non-coding regions, *i.e*. introns and flanking regions [32-36]. Recent attempts to disregard the pivotal role of the GC3 parameter in understanding the genome organization [37], failed to take into consideration that "the use of indirect methods can lead to apparently conflicting conclusions" [38]. Recently, the role of the GC3 parameter as genome marker was further confirmed by the unexpected finding of correlations with genome size and body mass of mammals [39]. The subset of KOG human genes analyzed in the present paper followed, as expected, the well assessed rules first described in the 90's [32-36]. These rules held not only for the whole set of genes, but also when the genes were grouped in the KOG functional classes (see Additional file 8 for statistical reports).

In order to shed light on the debate around the evolutionary forces shaping the base composition among and within genomes [4,6-10,16-18], genes were classified in the three functional categories [namely: (i) information storage and processing (Blue); (ii) cellular processes and signaling (Black); (iii) metabolism (Red)] and their base compositional properties were analyzed. The results showed that within mammalian genomes the three functional categories were characterized by a different GC3 content, following the pattern Blue < Black < Red (Figure 2 and 3). It is worth to stress that, regarding platypus and opossum, no significant differences were observed comparing the Black vs. the Blue category (see Additional file 1). No pattern was found in the reptile and amphibian genomes (Figure 2).

### Do current hypotheses explain the above finding?

It is worth to bring to mind that the keystone of the biased gene conversion hypothesis (BGC) was the strong correlation between hot spot recombination sites and GC content, establishing a cause/effect link of the first over the second parameter [7]. Consequently, the genomic impact of the BGC would be an increment of the GC content detectable at non-synonymous sites, synonymous sites, flanking and intronic sequences [9]. Since the BGC was reported to mimic perfectly natural selection [9], the compositional correlations holding in the human genome [32-36], including those reported in Additional file 8 could not be considered evidences for natural selection hypothesis.

In the light of the BGC hypothesis, the Blue < Black < Red pattern (observed in the majority of mammalian genomes) could have been explained as the result of the star-like phylogeny of mammals [40]. However, comparative genome analyses showed that hot spot recombination sites are "highly mobile" and therefore not phylogenetically related [41]. A result further supported by the studies conducted on the fast-evolving DNA-binding domain of PRDM9, identified as a major hotspot determinant of recombination. Indeed, the sequences and the number of PRDM9 domains were reported to vary a lot among species (reviewed in [42]), The lack of the Blue < Black < Red pattern in both frog and lizard and its appearance in mammalian genomes at present stands unclear. Although BGC received support from the analysis of the short sequences HARs and HACNSs in the human genome [43], considering that BGC was reported to be a widespread process affecting all genomes [9], the hypothesis was unable to explain the base compositional variability among bacterial genomes [44].

An interesting alternative hypothesis to the BGC was that proposed by Lemaitre and colleagues based on the analysis of the DNA breakpoint regions (BPR) [10]. Very recently, indeed, a 3D analysis of BPR showed that "two loci distant in the human genome but adjacent in the mouse genome are significantly more often observed in close proximity in the human nucleus than expected" [45]. The conservation of the Blue < Black < Red pattern among mammals, that started to diverge about 100 Mya [40], could probably be explained by the fact that 3D chromatin structure could be conserved over long evolutionary distances [45]. The time of divergence between amniotes and amphibian and between mammals and lizard was estimated to be several orders of magnitude greater than that of mammalian radiation (340-370Mya [46] and ~310Mya [40], respectively). Therefore explaining why the pattern was not conserved in reptiles and amphibians. However, according to the BPR hypothesis, evolutionary rearrangement breakages happen with a uniform propensity along the genome [10], leaving unexplained how the Blue < Black < Red pattern, absent in frog and lizard, could have been evolved in mammals. Moreover, as far as we know, no evidence has been produced to explain the base compositional variability among bacterial genomes in the light of the BPR hypothesis.

The critical query (Blue < Black < Red pattern) could be explained, on the contrary, by both thermal stability and metabolic rate hypotheses [16-18]. Indeed, *in situ *hybridization experiments performed on both human and amphibian nuclei (*i.e. Rana esculenta*), showed a comparable chromatin organization [47,48]. In both genomes, GC-poorest regions were found in closed chromatin structures localized at the nuclear periphery, while GC-richest ones were found in open chromatin structures localized more internally the nuclei [47,48]. According to the above reports, the different living temperature experienced by amphibians and mammals, could induce an increment of the GC content in mammals, in order to stabilize the open chromatin structures [16]. On the other hand, an increment of the metabolic rate, well known to be higher in mammals, should induce an increment of the GC content to increase DNA bendability, on one hand, and decrease nucleosome formation potential, on the other, to face an increment of transcriptional activity [17,18]. To this regard it should be recalled that along human chromosome the GC content and the gene expression profiles showed a positive correlation [19].

Temperature and metabolic rate are well known to be strongly correlated [49]. Therefore, disentangle the two variables would be not an easy task in the light of present data, also considering that terrestrial animals are living in an environment where oxygen is not a limiting factor. The problem was recently addressed analyzing the genomes of organisms living in aquatic habitats were the available oxygen in the environment is limited by the Henry's law. The analyses of teleostean fish genomes showed that: i) the genomic GC content of polar fish was higher than that of tropical fish; ii) that a positive and significant correlation holds between GC content and metabolic rate; and iii) a negative correlation was found between environmental temperature and GC content [50,51]. The problem was tackled in the present paper analyzing the orthologous pairs of human/frog (H/F) and human/lizard (H/L) genes. In both cases, the highest &#916GC3 turned out to take place in the Red category, that is the functional category grouping genes involved in metabolic processes (Figure 4). Although not resolving the dichotomy between temperature and metabolic rate (both increasing, indeed, from frog to human [52]) the result was congruent with the conclusion drawn out from the comparison of teleostean fish genomes [50,51].

The detailed investigation on the distribution of the KOG functional classes revealed that the Blue < Black < Red pattern was even more multifaceted. Indeed, in the positive side of the human butterfly plot, apart the majority of Red bars, the B and K blue bars, as well as the T and Z black bars were also observed (Figure 5, panel C). The above picture was not confined to the human genome, but commonly found in all mammals. Indeed, the B and T classes were in the positive side of the butterfly plot in the 93% of the cases, whereas the K and Z classes reached the 100% of the cases (Figure 5, panel C). The occurrence of the bars belonging to the Red category ranged from 86% of the Q class to 100% of the G, E and P classes. Needless to say, the pattern was not found in the frog and lizard genomes. All the considerations formerly drawn out in the light of the different evolutionary hypotheses regarding the Blue < Black < Red pattern, applied even more radically to the pattern of functional classes clustering in the positive side of all mammalian butterfly plots (Additional files 3, 4, 5, 6, 7 and Figure 5, panel C), showing a different chromosomal distribution.

The above result deserves a more detailed argumentation. As reported in Table 1 the genes belonging to the four classes were involved in the following task: Chromatin structure and dynamics (B), Transcription (K), Signal transduction mechanisms (T) and Cytoskeleton (Z). The fact that the GC3 content of genes belonging to the B and K classes was not surprising, since an increment of the metabolic rate affects transcription process and chromatin structure, as discussed above. More inscrutable was the result regarding the T and Z classes. Recently, an interesting paper was published on the effect of estrogen exposure in mice brain, inducing an increment of the expression level of a discrete number of genes [53]. From their results it is possible to derive that beside a 39% of genes involved in metabolic processes, 18% belonged to the Z class and 25% to the T one, whereas only 6% of the genes belonged to the category grouping genes involved in information storage and processing. Szego's and colleagues report [53] was an interesting preliminary approach, pointing towards further investigations on the link between genome organization and the physiological reaction to stressing stimuli increasing the metabolic rate. Interestingly, gene clusters for metabolic pathways have been reported also in plants (reviewed in [54]).

## Conclusions

All the different evolutionary hypotheses proposed till now surely contribute, with different weight, to the compositional variability observed among and within organisms [55]. Few, however, seem to fit with the very wide range from prokaryotes to eukaryotes. Indeed, recent analysis showed that mutational bias cannot explain genome composition in bacteria, reviewed in [56]. The BGC hypothesis, supported by the data produced on sequences HARs and HACNSs in mammals [43], also failed to explain the base compositional variability among bacterial genomes [44], and hardly explains the present results. The BPR hypothesis was very promising, especially in the light of the studies carried out on conservation of the 3D chromatin structure over long evolutionary distances [45]. However, still remain to clarify the mechanism leading to the observed patterns in mammals.

Regarding the thermodynamic hypothesis, the extensive studies carried out on bacterial genomes has been matter of debate [57-60]. Within bacterial families a significant positive correlation between growth temperature and GC content was observed in 9 out of 20 families [58]. However, the positive correlation failed to be observed in teleostean fish genomes, where a negative one was found indeed [50,51]. Unfortunately, present data neither shed light in favor nor against the effect of temperature on the compositional transition from amphibian/reptile to mammals [24-26]. On the contrary, the metabolic rate hypothesis [17,18] not only explained both the transition [61] and the shifting mode of evolution of vertebrate genomes [50,51], but also the within genome patterns showed in the present paper. Moreover, a correlation between metabolic rate and GC % has been found also in bacteria [12,15], as well as among teleostean fish [50,51]. It is worth to bring to mind that, although the metabolic rate hypothesis is in the frame of the adaptive hypotheses, most probably there is no need to invoke the effect of the positive selection. Indeed, the shift of the of threshold for the "best-fit GC content" could account for the genome compositional shift observed comparing teleostean fish living in different habitats [50]. Natural selection has been also proposed to explain the great compositional heterogeneity of the human genome [62].

## Abbreviations

BGC: biased gene conversion; bp: base pair; BPR: DNA breakpoint distribution; BRH: best reciprocal hit; GC: molar ratio of guanine plus cytosine; GC1 + 2: molar ratio of guanine plus cytosine at first and second positions; GC3: molar ratio of guanine plus cytosine at third codon positions; ΔGC3: GC3 increment; GCi: molar ratio of guanine plus cytosine in intronic sequences; HACNS: human-accelerated conserved non-coding sequences; HAR: human-accelerated region; KOG: clusters of orthologous groups for eukaryotic complete genomes.

## Competing interests

The authors declare that they have no competing interests.

## Authors' contributions

LB carried out KOG analysis and drafted the manuscript. AC retrieved and analyzed non-coding sequences. CA performed and described statistical analyses. CF assigned CDS to the chromosomal band types. SS contributed to the design of the work and to the general discussion. GD envisages the design and coordination of the study and wrote the manuscript. All authors read and approved the final manuscript.

## Supplementary Material

Additional file 1 **The de Finetti's diagram**. Click here for file

Additional file 2 ***p*-values of the Mann-Whitney test among categories**. Click here for file

Additional file 3 **Butterfly plot of mammalian order: primates**. Click here for file

Additional file 4 **Butterfly plot of mammalian order: rodentia and lagomorpha**. Click here for file

Additional file 5 **Butterfly plot of mammalian order: artiodactyla, perissodactyla, chiroptera, cetacea**. Click here for file

Additional file 6 **Butterfly plot of mammalian order: didelphimorpha and monotremata**. Click here for file

Additional file 7 **Butterfly plot of mammalian order: proboscidea and cingulata**. Click here for file

Additional file 8 **Statistical Summary of KOG genes**. Click here for file
