# Supplementary material for: The footprint of metabolism in the organization of mammalian genomes
Source: BMC Genomics. 2012 May 8;13:174. doi: 10.1186/1471-2164-13-174 (PMC3384468; doi:10.1186/1471-2164-13-174)
Supplement: Additional file 1 — The de Finetti's diagram. [file 1471-2164-13-174-S1.PDF]

de Finetti's diagram: In order to assess the compositional/spatial distribution of the average GC3 in the three categories *c* (*c*= Blue, Black and Red) and compare such behavior across different organisms $g = 1, \ldots, G$, the whole GC3 range $[a_g, b_g]$ of each organism g was split in three equal size intervals, corresponding to the levels denoted as Low, Medium and High, respectively. Then for each organism *g* and each category *c* we defined the vector $\left(c_g^L, c_g^M, c_g^H\right)$ containing the normalized occurrency for the corresponding functional classes in the three levels. Clearly, we have $c_g^i \geq 0$ and $\sum_{i \in \{Low, Medium, high\}} c_g^i = 1$ for all organisms *g*. Since each vector $\left(c_g^L, c_g^M, c_g^H\right)$ can be represented as a point (whose color correspond to the category) in a de Finetti's diagram, each organism can be coded in the diagram using three colored points drawn in correspondence of its $\left\{\left(c_g^L, c_g^M, c_g^H\right)\right\}_{c \in \{blue, black, red\}}$ values. The de Finetti's diagram is a well known representation used in population genetics to show the range of genotype frequencies for which Hardy-Weinberg equilibrium is satisfied. Here we use it for comparing the GC3 compositional/spatial distribution between the categories in different organisms. Hence, to understand its meaning in our context we recall the Viviani's theorem that assures that sum of the distances from an internal point to the sides of an equilateral triangle equals the length of the triangle's altitude (that in our context is set to 1). According to such results each $\left(c_g^L, c_g^M, c_g^H\right)$ value can be represented as a point inside the triangle and the distances to the corresponding side is equal to c_g^i. In practice the closer one point is to a particular side, the lesser such category is present in that genome at the level showed in that side. Additionally, by dividing the area of the triangle with the three triangle's

altitudes 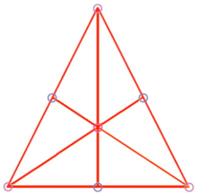 and considering the centroid we can define the three sectors (identified by the Low, Medium, High line) of the triangle having different $\left(c_g^L, c_g^M, c_g^H\right)$ relational ordering and hence different CG3 abundance. Categories belonging to a given sector show minimal presence of that CG3 level with respect to the other levels.

Intuitively, we observe that, in absence of association between the GC3 distribution andthe functional categories, each configuration of the vector $\left(c_g^L, c_g^M, c_g^H\right)$ is equally likely and, as a consequence, the different sectors are expected to be equally represented. Discrepancy from such uniform distribution denotes a specific association. To measure such effect, first we observed that, due to the finite number of classes in each category only a finite number of configurations results attainable. Such configurations are invariants with respect to 2/3π rotations of the triangle. However, due to the fact that the range $[a_g, b_g]$ is organism's specific the observed $\left\{c_g^L, c_g^M, c_g^H\right\}_{c \in \{blue, black, red\}}$ are not independent since at least one class should be present in either the low and high levels, hence the number of admissible configurations results less than the attainable.

The $\left\{c_g^L, c_g^M, c_g^H\right\}_{c \in \{blue, black, red\}}$ for each organism g are shown in Figure 1 where the value of each points represents the number of times the vector $\left(c_g^L, c_g^M, c_g^H\right)$ has been observed in the different organisms $g = 1, \ldots, G$. The de Finetti's diagram clearly showed that in the large majority mammalian genomes the Red category was confined to a restricted part of the space of the diagram (i.e., closed to the Low level line, denoting that in the large majority mammalian genomes the Red category: was rarely present in the lowest GC3

range. In order to test whether it was possible to obtain such configuration by chance, performed B class permutations among the categories and each time we counted k_i the occurrence of the Red class in the Low sector, then we estimated the p-value of the sector as $\frac{\sum_{i=1}^{B} I\left(k_i \geq k^*\right)}{B}$ where B denotes the number of permutation and k^* the observed occurrence of the Red class in the Low sector on our dataset.
