# Supplementary material for: The footprint of metabolism in the organization of mammalian genomes
Source: BMC Genomics. 2012 May 8;13:174. doi: 10.1186/1471-2164-13-174 (PMC3384468; doi:10.1186/1471-2164-13-174)
Supplement: Additional file 2 — p-values of the Mann-Whitney test among categories. [file 1471-2164-13-174-S2.PDF]

S2. *p*-values of the Mann-Whitney test among categories.

Class	Order	Species	Red *vs.* Blue	Red *vs.* Black	Black *vs.* Blue
**Mammals**	**Primates**	*H. sapiens*	hs	hs	hs
		G. Gorilla	hs	0.0027	0.0012
		P. pygmaeus	hs	0.0001	0.03
	**Rodents**	*M. musculus*	hs	0.0004	0.0006
		O. cuniculus	hs	0.0001	0.0005
		S. tridecemilineatus	hs	0.0022	hs
	**Laurasiatheria**	*B. taurus*	hs	0.107	0.0002
		E. caballus	hs	0.0002	0.0023
		P. vampyrus	hs	0.0276	0.0005
		T. truncatus	hs	0.0012	0.0028
	**Afrotheria**	*L. africana*	0.0002	0.0101	0.0916
	**Xenarthra**	*D. novemcinctus*	hs	0.0001	0.0002
	**Marsupials**	*O. anatinus*	0.0958	0.0224	0.7639
	**Monotremes**	*M. domestica*	0.0015	0.005	0.3252
**Reptiles**	**Squamata**	*A. cardiensis*	0.6258	0.1748	0.0625
**Amphibians**	**Anura**	*X. tropicalis*	0.4994	0.8865	0.5715

hs = Highly significant p-value lower than at least 10^{-5}
