# Supplementary figures and images for: The footprint of metabolism in the organization of mammalian genomes
Source: BMC Genomics. 2012 May 8;13:174. doi: 10.1186/1471-2164-13-174 (PMC3384468; doi:10.1186/1471-2164-13-174)

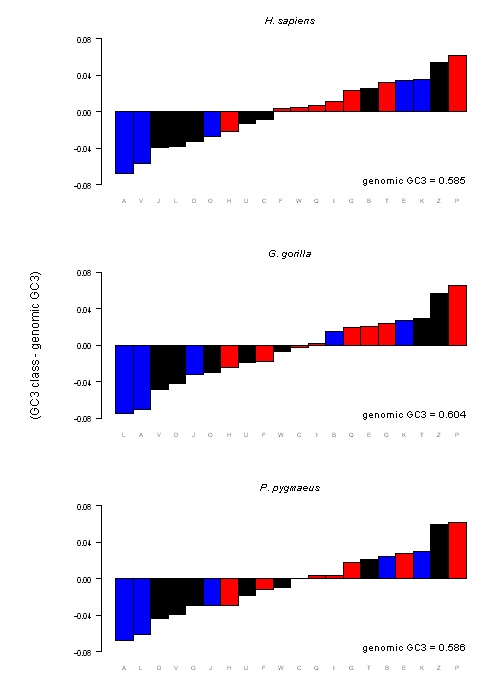

Supplement: Additional file 3 — Butterfly plot of mammalian order: primates. [file 1471-2164-13-174-S3.JPEG]

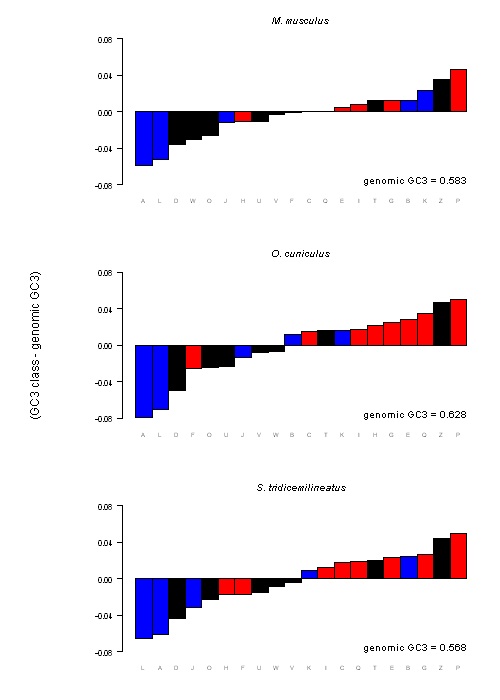

Supplement: Additional file 4 — Butterfly plot of mammalian order: rodentia and lagomorpha. [file 1471-2164-13-174-S4.JPEG]

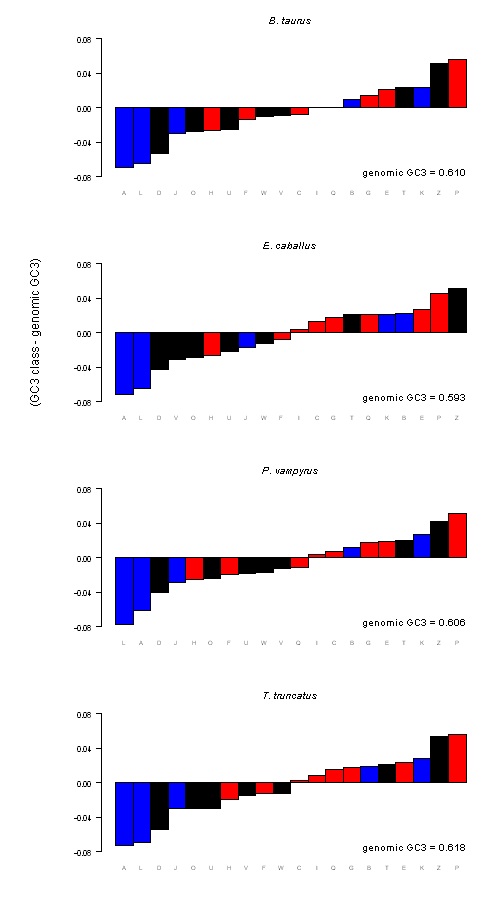

Supplement: Additional file 5 — Butterfly plot of mammalian order: artiodactyla, perissodactyla, chiroptera, cetacea. [file 1471-2164-13-174-S5.JPEG]

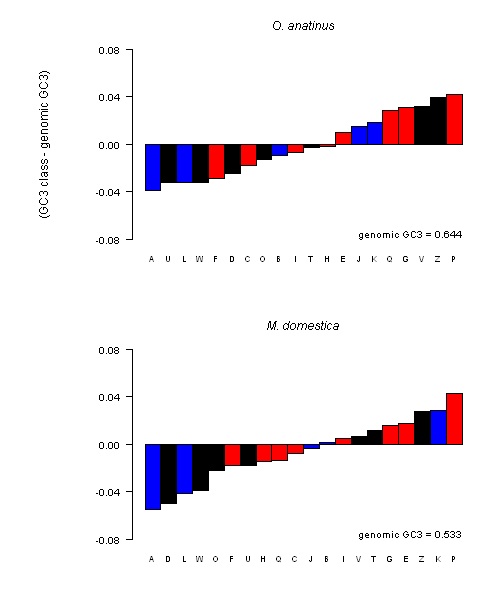

Supplement: Additional file 6 — Butterfly plot of mammalian order: didelphimorpha and monotremata. [file 1471-2164-13-174-S6.JPEG]

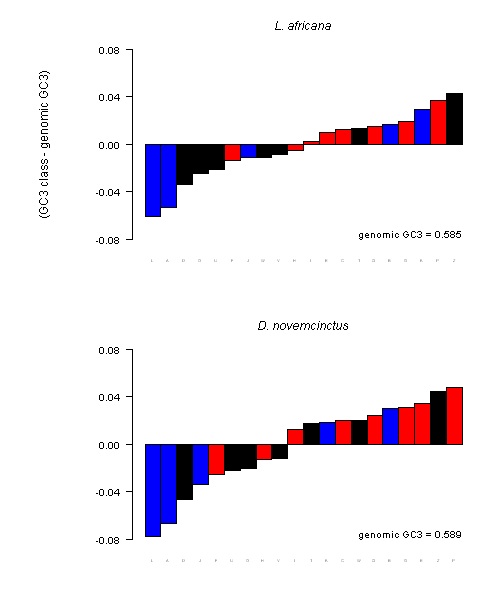

Supplement: Additional file 7 — Butterfly plot of mammalian order: proboscidea and cingulata. [file 1471-2164-13-174-S7.JPEG]
