# Supplementary material for: The footprint of metabolism in the organization of mammalian genomes
Source: BMC Genomics. 2012 May 8;13:174. doi: 10.1186/1471-2164-13-174 (PMC3384468; doi:10.1186/1471-2164-13-174)
Supplement: Additional file 8 — Statistical Summary of KOG genes. [file 1471-2164-13-174-S8.PDF]

KOG genes

Regression Coefficient of GC12 *vs.* GC3, %

|R| 0.589 - R Squared 0.347

	Coefficient	Std. Error	t-Value	P-Value
Intercept	36.099	0.141	255.186	<0.0001
GC3, %	0.214	0.002	91.374	<0.0001

Note: genes of the functional class **W** were removed according to the analysis of the outliers (http://home.ubalt.edu/ntsbarsh/Business-stat/otherapplets/Outlier.htm), in agreement with the results of reference [29].

Regression Coefficient of GCi *vs.* GC3, %

|R| 0.805 - R Squared 0.648

	Coefficient	Std. Error	t-Value	P-Value
Intercept	0.241	0.002	127.281	<0.0001
GC3, %	0.384	0.003	125.330	<0.0001

Regression Coefficient of 3′-Flanking GC *vs.* GC3, %

|R| 0.761 - R Squared 0.579

	Coefficient	Std. Error	t-Value	P-Value
Intercept	0.209	0.002	85.904	<0.0001
GC3, %	0.428	0.004	108.463	<0.0001

Regression Coefficient of 5′-Flanking GC *vs.* GC3, %

|R| 0.533 - R Squared 0.284

	Coefficient	Std. Error	t-Value	P-Value
Intercept	0.352	0.003	119.758	<0.0001
GC3, %	0.276	0.005	58.187	<0.0001

KOG Functional Classes

Regression Coefficient of GC12 *vs.* GC3, %

|R| 0.469 - R Squared 0.220

	Coefficient	Std. Error	t-Value	P-Value
Intercept	39.616	3.817	10.379	<0.0001
GC3, %	0.223	0.065	2.314	<0.0320

Note: functional class W was removed according to

Regression Coefficient of GCi *vs.* GC3, %

|R| 0.819 - R Squared 0.671

	Coefficient	Std. Error	t-Value	P-Value
Intercept	0.338	0.022	15.560	<0.0001
GC3, %	0.223	0.037	6.061	<0.0001

Regression Coefficient of 3′-Flanking GC *vs.* GC3, %

|R| 0.678 - R Squared 0.460

	Coefficient	Std. Error	t-Value	P-Value
Intercept	0.351	0.029	12.293	<0.0001
GC3, %	0.189	0.048	3.913	0.0010

Regression Coefficient of 5′-Flanking GC *vs.* GC3, %

|R| 0.298 - R Squared 0.089

	Coefficient	Std. Error	t-Value	P-Value
Intercept	0.461	0.041	11.176	<0.0001
GC3, %	0.092	0.070	1.325	0.2016
